# Approaches to informed consent for hypothesis-testing and hypothesis-generating clinical genomics research

**DOI:** 10.1186/1755-8794-5-45

**Published:** 2012-10-10

**Authors:** Flavia M Facio, Julie C Sapp, Amy Linn, Leslie G Biesecker

**Affiliations:** 1 National Human Genome Research Institute, National Institutes of Health, Bethesda, MD, USA; 2 current affiliation: Kennedy Krieger Institute, Baltimore, MD, USA

**Keywords:** Whole genome sequencing, Whole exome sequencing, Informed consent

## Abstract

**Background:**

Massively-parallel sequencing (MPS) technologies create challenges for informed consent of research participants given the enormous scale of the data and the wide range of potential results.

**Discussion:**

We propose that the consent process in these studies be based on whether they use MPS to test a hypothesis or to generate hypotheses. To demonstrate the differences in these approaches to informed consent, we describe the consent processes for two MPS studies. The purpose of our hypothesis-testing study is to elucidate the etiology of rare phenotypes using MPS. The purpose of our hypothesis-generating study is to test the feasibility of using MPS to generate clinical hypotheses, and to approach the return of results as an experimental manipulation. Issues to consider in both designs include: volume and nature of the potential results, primary versus secondary results, return of individual results, duty to warn, length of interaction, target population, and privacy and confidentiality.

**Summary:**

The categorization of MPS studies as hypothesis-testing versus hypothesis-generating can help to clarify the issue of so-called incidental or secondary results for the consent process, and aid the communication of the research goals to study participants.

## Background

Advances in DNA sequencing technologies and concomitant cost reductions have made the use of massively-parallel sequencing (MPS) in clinical research practicable for many researchers. Implementations of MPS include whole genome sequencing and whole exome sequencing, which we consider to be the same, for the purposes of informed consent. A challenge for researchers employing these technologies is to develop appropriate informed consent [1,2], given the enormous amount of information generated for each research participant, and the wide range of medically-relevant genetic results. Most of the informed consent challenges raised by MPS are not novel – what is novel is the scale and scope of genetic interrogation, and the opportunity to develop novel clinical research paradigms.

Massively-parallel sequencing has the capacity to detect nearly any disease-causing gene variant, including late-onset disorders, such as neurologic or cancer-susceptibility syndromes, subclinical disease or endo-phenotypes, such as impaired fasting glucose, and heterozygous carriers of traits inherited in a recessive pattern. Not only is the range of the disorders broad, but the variants have a wide range of relative risks from very high to nearly zero. This is a key distinction of MPS when compared to common SNP variant detection (using so-called gene chips). Because some variants discovered by MPS can be highly penetrant, the detection of such variants can have enormous medical and counseling impact. While many of these informed consent issues have been addressed previously [1,3], the use of MPS in clinical research combines these issues and is on a scale that is orders of magnitude greater than previous study designs.

The initial clinical research uses of MPS were a brute force approach to the identification of mutations for rare mendelian disorders [4]. This is a variation of positional cloning (also known as gene mapping) and thus a form of classical hypothesis-testing research. The hypothesis is that the phenotype under study is caused by a genetic variant and a suite of techniques is employed (in this case MPS) to identify that causative variant. The application of this technology in this setting is of great promise and will identify causative gene variants for numerous traits, with some predicting that the majority of Mendelian disorders will be elucidated in 5–10 years.

The second of these pathways to discovery is a more novel approach of generating and then sifting MPS results as the raw material to allow the generation of clinical hypotheses, which are in turn used to design clinical experiments to discover the phenotype that is associated with that genotype. This approach we term hypothesis-generating clinical genomics. These hypothesis-generating studies require a consent process that provides the participant with an understanding of scale and scope of the interrogation, which is based on a contextual understanding of the goal and overall organization of the research since specific risks and benefits can be difficult to delineate [5,6]. Importantly, participants need to understand the notion that the researcher is exploring their genomes in an open-ended fashion, that the goal of the experiment is not predictable at the outset, and that the participant will be presented with downstream situations that are not currently foreseeable.

We outline here our approaches to informed consent for our hypothesis-testing and hypothesis-generating MPS research studies. We propose that the consent process be tailored depending on which of these two designs is used, and whether the research aims include study of the return of results.

### General issues regarding return of results

Participants in our protocols have the option to learn their potentially clinically relevant genetic variant results. The issue of return of results is controversial and the theoretical arguments for and against the return of results have been extensively debated [7]. Although an increasing body of literature describes the approaches taken by a few groups no clear consensus exists in either the clinical genomics or bioethics community [8]. At one end of the spectrum there are those who argue that no results should be returned [9], and at the other end others contend that the entire sequence should be presented to the research participant [10-12]. In between these extremes lies a qualified or intermediate disclosure policy [13,14]. We take the intermediate position in both of our protocols by giving research participants the choice to receive results, including variants deemed to be clinically actionable [3,15]. Additionally, both protocols are investigating participants’ intentions towards receiving different types of results in order to inform the disclosure policies within the projects and in the broader community [16]. Because one of our research goals is to study the issues surrounding return of results, it is appropriate and necessary to return results. Thus, the following discussion focuses on issues pertinent to studies that plan to return results.

### Issues to consider

#### Issue #1: Primary versus secondary variant results and the open-ended nature of clinical genomics

In our hypothesis-testing study we distinguish variants as either primary or secondary variants, the distinction reflecting the purpose of the study. A primary variant is a mutation that causes the phenotype that is under study, i.e., the hypothesis that is being tested in the study. A secondary variant is any mutation result not related to the disorder under study, but discovered as part of the quest for the primary variant.

We prefer the term ‘secondary’ to ‘incidental’ because the latter is an adjective indicating chance occurrence, and the discovery of a disease causing mutation by MPS cannot be considered a chance occurrence. The word ‘incidental’ also suggests a lesser degree of importance or impact and it is important to recognize that secondary findings can be of greater medical or personal impact than primary findings.

The consent discussion about results potentially available from participation in a hypothesis-testing study is framed in terms of the study goal, and we assume a high degree of alignment between participants’ goals and the researchers’ aims with respect to primary variants. Participants are, in general, highly motivated to learn the primary variant result and we presume that this motivation contributed to their decision to enroll in the study, similar to motivations for those who have been involved in positional cloning studies. This motivation may not hold for secondary variants, but our approach is to offer them the opportunity to learn secondary and actionable variants that may substantially alter susceptibility to, or reproductive risk for, disease.

In the hypothesis-generating study design no categorical distinction (primary vs. secondary) is made among pathogenic variants, i.e., all variants are treated the same without the label of ‘primary’ or ‘secondary’. This is because we are not using MPS to uncover genetic variants for a specific disease, and any of the variants could potentially be used for hypothesis generation. We suggest that this is the most novel issue with respect to informed consent as the study is open-ended regarding its goals and downstream research activities. This is challenging for informed consent because it is impossible to know what types of hypotheses may be generated at the time of enrollment and consent.

Because the downstream research topics and activities are impossible to predict in hypothesis-generating research, subjects must be consented initially to the open-ended nature of the project. During the course of the study, they must be iteratively re-consented as hypothesis are generated from the genomic data and more specific follow-up studies are designed and proposed to test those newly generated hypotheses. These downstream, iterative consents will vary in their formality, and the degree to which they need to be reviewed and approved. Some general procedures can be approved in advance; for example it may be anticipated that segregation studies would be useful to determine causality for sequence variants or the investigator may simply wish to obtain some additional targeted medical or family history from the research subject. This could be approved prospectively by the IRB with the iterative consent with the subject comprising a verbal discussion of the nature of the trait for which the segregation analysis or additional information is being sought. More specific or more invasive or risky iterative analyses would necessitate review and approval by the IRB with written informed consent.

##### Informed consent approach

The informed consent process must reflect the fundamental study design distinction of hypothesis-testing versus hypothesis-generating clinical genomics research. For the latter, the challenge is to help the research subjects understand that they are enrolling in a study that could lead to innumerable downstream research activities and goals. The informed consent process must be, like the research, iterative, and involve ongoing communication and consent with respect to those downstream activities.

#### Issue #2: Volume and nature of information

Whole genome sequencing can elucidate an enormous number of variations for a given individual. A typical whole genome sequence yields ~4,000,000 sequence variations. A whole exome sequence limits the interrogation to the coding regions of genes (about 1–1.5% of the genome) and generates typically 30,000-50,000 gene variants. While most are benign or of unknown consequence, some are associated with a significant increased risk of disease for the individual and/or their family members. For example, the typical human is a carrier for three to five deleterious genetic variants or mutations that cause severe recessive diseases [17,18]. In addition, there are over 30 known cancer susceptibility syndromes, which in aggregate may affect more than 1/500 patients, and the sequence variants that cause these disorders can be readily detected with MPS. These variants can have extremely high relative risks. For some disorders, a rare variant can be associated with a relative risk of greater than 1,000. This is in contrast with common SNP typing which detects variants associated with small relative risks (typically on the order of 1.2-1.5). It is arguable whether the latter type of variant has any clinical utility as an individual test.

Conveying the full scope of genomic interrogation planned for each sample and the volume of information generated for a given participant is impossible. The goal and challenge in this instance is to give the participant as realistic a picture as possible of the likely amount of clinically actionable results the technology can generate. Our approach is two-fold: to give the subjects the clear message that the number and nature of the findings is enormous and literally impossible to describe in a comprehensive manner and to use illustrative examples of the spectrum of these results.

To provide examples, we bin genetic variants into broad categories, as follows: heterozygous carriers of genetic variants implicated in recessive conditions (e.g., *CFTR* p.Phe508del and cystic fibrosis); variants that cause a treatable disorder that may be present, but asymptomatic or undiagnosed (e.g., *LDLR* p.Trp87X, familial hypercholesterolemia); variants that predispose to later-onset conditions (e.g., *BRCA2* c.5946delT (commonly known as c.6174delT), breast and ovarian cancer susceptibility); variants that predispose to late-onset but untreatable disorders (e.g., frontotemporal dementia *MAPT* p.Pro301Leu).

Additionally, the scale and scope of the results determines a near certainty that all participants will be found to harbor disease-causing mutations. This is because the interrogation of all genes brings to light the fact that the average human carries 3–5 recessive deleterious genes in addition to the risks for later onset or incompletely penetrant dominant disorders. This reality can be unsettling and surprising to research subjects and we believe it is important to address this early in the process, not downstream in the iterative phase. It is essential for the participants to choose whether MPS research is appropriate for them, taking into account their personal views and values.

##### Informed consent approach

Communicate to participants both the overwhelming scale and scope of genetic results they may opt to receive and provide them with specific disease examples that illustrate the kinds of decisions they may need to make as the results become available. These examples should also assist the research subjects in making a decision about whether to participate in the study and if so, the kinds of decisions they may need be making in the future as results become available.

#### Issue #3: Return of individual genotype results

The return of individual genotype results from MPS presents a new challenge in the clinical research environment, again because of the scale and breadth of the results. The genetic and medical counseling can be challenging because of the volume of results generated, participants’ expectations, the many different categories of results, and the length of time for the information to be available. We suggest that the most reasonable practice is to take a conservative approach and disclose only clinically actionable results. To this end, the absence of a deleterious gene variant (or a negative result) would not be disclosed to research participants. It is our understanding that it is mandatory to validate any individual results that are returned to research subjects in a CLIA-certified laboratory. Using current clinical practice as a standard or benchmark, we suggest that until other approaches are shown to be appropriate and effective, disclosure should take place during a face-to-face encounter involving a multidisciplinary team (geneticist, genetic counselor, and specialists on an ad-hoc basis based on the phenotype in question).

During the initial consent, participants are alerted to the fact that in the future the study team will contact them by telephone and their previously-stated preferences and impressions about receiving primary and secondary variant results will be reviewed. The logistics and details of this future conversation feature prominently in the initial informed consent session, as it is challenging to make and to receive such calls. Participants make a choice to learn or not learn a result each time a result becomes available. Once a participant makes the decision to learn a genotype result, the variant is confirmed in a CLIA lab, and a report is generated. The results are communicated to the participant during a face-to-face meeting with a geneticist and genetic counselor, and with the participation of other specialists depending on the case and the participant’s preferences. These phone discussions are seen as an extension of the initial informed consent process and as opportunities for the participants to make decisions in a more relevant and current context (compared to the original informed consent session). We see this as an iterative approach to consent, also known as circular consent [5]. Participants who opt not to learn a specific result can still be contacted later if other results become available, unless they choose not to be contacted by us any longer.

This approach to returning results is challenged by the hypothesis-generating genomics research approach. Participants in our hypothesis-testing protocol are not asked to make a decision about learning individual genotype results at the time of consent. This is because we cannot know the nature of the future potential finding at the time of the original consent. Rather, they are engaged in a discussion of what they currently imagine their preferences might be at some future date, again using exemplar disorders and hypothetical scenarios of hypothesis-generating studies.

In the hypothesis-generating study, we have distinct approaches for variants in known disease-causing genes versus variants in genes that are hypothesized to cause disease (the latter being the operative hypothesis generating activity). For the former, the results are handled in a manner quite similar to the hypothesis-testing study. In the latter case, the participant may be asked if they would be willing to return for further phenotyping to help us determine the nature of the variant of uncertain clinical significance (VUCS). The participant is typically informed that they have a sequence variant and that we would like to learn, through clinical research whether this variant has any phenotypic or clinical significance. It is emphasized that current knowledge does not show that the variant causes any phenotype and the chances are high that the variant is benign. However, neither the gene nor the sequence variant is disclosed and the research finding is not confirmed in a CLIA certified lab. This type of VUCS would only be communicated back to the participant if the clinical research showed that the variant was causative, and the return of the result was determined medically appropriate by our Mutation Advisory Committee, and following confirmation in a CLIA-certified laboratory.

##### Informed consent approach

For the return of mutations in known, disease causing genes, the initial consent cannot comprehensively inform subjects of the nature of the diseases, because of the scale and scope of the potential results. Instead, exemplars are given to elicit general preferences, which are then affirmed or refined at the time results are available. Hypothesis-generating studies require that subjects receive sufficient information to make an informed choice about participation in the specific follow-up study, with return of individual results only if the cause and effect relationship is established, with appropriate oversight.

#### Issue #4: Duty to warn

Given the breadth of MPS gene interrogation, it is reasonable to anticipate that occasional participants may have mutations that pose a likely severe negative consequence, which we classify as “panic” results. This models clinical and research practice for the return of results such as a pulmonary mass or high serum potassium level. In contrast to the above-mentioned autosomal recessive carrier states that are expected to be nearly universal, genetic panic results should be uncommon. However, they should not be considered as unanticipated – it is obvious that such variants will be detected and the informed consent process should anticipate these. Examples would be deleterious variants for malignant hyperthermia or Long QT Syndrome, either of which have a substantial risk of sudden death and the risk can be mitigated.

Both our hypothesis-testing and hypothesis-generating studies include mechanisms for the participants to indicate the types of results that they wish to have returned to them. In the hypothesis-testing mode of research this is primarily to respect the autonomy of the participants, but in addition, for the hypothesis-generating study we are assessing the motivations and interests of the subjects in various types of results and manipulating the return of results as an experimental aim. It is our clinical research experience that participants are challenged by making decisions regarding possible future results that are rare, but potentially severe. As well, the medical and social contexts of the subjects evolves over time and the consent that was obtained at enrollment may not be relevant or appropriate at a later time when such a result arises. This is particularly relevant for a research study that is ongoing for substantial periods of time (see also point #7, below).

To address these issues we have consented the subjects to the potential return of “panic” results, irrespective of their preferences at the initial consent session. In effect, the consent process is for some participants a consent to override their preference.

##### Informed consent approach

In both hypothesis-testing and hypothesis-generating research it is important to outline circumstances in which researchers’ duty-to-warn may result in a return of results that may be contrary to the preferences of the subject. It is essential that the subjects understand this approach to unusually severe mutation results. Subjects who are uncomfortable with this approach to return of results are encouraged to decline enrollment.

#### Issue #5: Length of researcher and participant interaction

Approaches to MPS data are evolving rapidly and it is anticipated that this ongoing research into the significance of DNA variants will continue for years or decades. The different purposes of the two study designs lead to different endpoints in terms of researcher’s responsibility to analyze results. In our hypothesis-testing research, discussion of the relationship of the participants to the researchers is framed in terms of the discovery of the primary variant. We ask participants to be willing to interact with us for a period of months or years as it is impossible for to set a specific timeline to determine the cause of the disorder under investigation (if it ever discovered). While attempts to elucidate the primary variant are underway, participants’ genomic data are periodically annotated using the most current bioinformatic methodologies available. We conceptualize our commitment to return re-annotated and updated results to participants as diminishing, but not disappearing, after this initial results’ disclosure. As the primary aim of the study has been accomplished, less attention will be directed to the characterization of ancillary genomic data, yet we believe we retain an obligation to share highly clinically actionable findings with participants should we obtain them.

In the hypothesis-generating study the researcher’s responsibility to annotate participants’ genomes/exomes is ongoing. This is ongoing because, as noted above, one of the experimental aims is to study the motivations and interests of the subjects in these types of results. Determining how this motivation and interest fares over time is an important research goal. During the informed consent discussion it is emphasized that the iterative nature of result interpretation will lead to multiple meetings for the disclosure of clinically actionable results, and that the participant may be contacted months or years after the date of enrollment. Additionally, it is outlined that the participant will make a choice about learning the result each time he/she is re-contacted about the availability of a research finding, and that finding will only be confirmed in a CLIA-certified laboratory if the participant opts to learn the information. Participants who return to discuss results are reminded that they will be contacted in the future if and when other results deemed to be clinically actionable are found for that individual.

##### Informed consent approach

Describe nature, mutual commitments, and duration of researcher-participant relationship to participants. For hypothesis-testing studies it is appropriate that the intensity of the clinical annotation of secondary variants may decline when the primary goal of the study is met. For hypothesis-generating studies, such interactions may continue for as long as there are variants to be further evaluated and as long as the subject retains an interest in the participation.

#### Issue #6: Target population

The informed consent process needs to take into account the target population in terms of their disease phenotype, age, and whether the goal is to enroll individual participants or families. These considerations represent the greatest divergence in approaches to informed consent when comparing hypothesis-testing and hypothesis-generating research. In our two studies, the hypothesis-testing study focuses on rare diseases and often family participation, whereas the hypothesis-generating study focuses on more common diseases and unrelated index cases. There are an infinite number of study designs and investigators may adapt our approaches to informed consent for their own designs.

Our hypothesis-testing protocol enrolls both individual participants and families (most commonly trios), the latter being more common. In hypothesis-testing research, many participants are either affected by a genetic disease or are a close relative (typically a parent) of a person with a genetic disease. The research participants must weigh their hope for, and personal meaning ascribed to, learning the genetic cause for their disorder against the possibility of being in a position to learn a significant amount of unanticipated information. Discussing and addressing the potential discrepancy of the participants’ expectations of the value of their results and what they may realistically stand to learn (both desired and undesired information) is a central component of the informed consent process.

In our hypothesis-testing protocol, when parents are consenting on behalf of a minor child, we review with them the issues surrounding genetic testing of children and discuss their attitudes regarding their child’s autonomy and their parental decision-making values. Because family trios (most often mother-father-child) are enrolled together, we discuss how one individual’s preferences regarding results may be disrupted or superseded by another family member’s choice and communication of that individual’s knowledge.

In contrast, our hypothesis-generating protocol enrolls as probands or primary participants older, unrelated individuals [19]. Most participants are self-selected in terms of their decision to enroll and are not enrolled because they or a relative have a rare disease. Participants in the hypothesis-generating protocol are consented for future exploration of any and all possible phenotypes. This is a key distinguishing feature of this hypothesis-generating approach to research, which is a different paradigm – going from genotype to phenotype. The participants may be invited for additional phenotyping. In fact, multiple satellite studies are ongoing to evaluate various subsets of participants for different phenotypes. The key with the consent for these subjects is to initially communicate to the subjects the general approach – that their genome will be explored, variations will be identified, and they may be re-contacted for a potential follow-up study to understand the potential relationship of that variant to their phenotype. These subsequent consents for follow-up studies are considered an iterative consent process, which is similar to the Informed Cohort concept [20].

##### Informed consent approach

Hypothesis-generating research is a novel approach to clinical research design and requires an ongoing, iterative approach to informed consent. For hypothesis-testing research a key informed consent issue is for the subjects to balance the desire for information on the primary disease causing mutation with the pros and cons of obtaining possibly undesired information on secondary variants.

#### Issue #7: Privacy and confidentiality

In MPS studies, privacy and confidentiality is a complex and multifaceted issue. Some potential challenges include: the deposition of genetic and phenotypic data in public databases, the placement of CLIA-validated results in the individual’s medical chart, and the discovery of secondary variants in relatives of affected probands in family-based (typically hypothesis-testing) research.

The field of genomics has a tradition of deposition of data in publicly accessible databases. Participants in our protocols are informed that the goal of sharing de-identified information in public databases is to advance research, and that there are methods in place maximize the privacy and confidentiality of personally identifiable information. However, the deposition of genomic-scale data for an individual participant, such as a MPS sequence, is far above the minimal amount of data to uniquely identify the sample [21,22]. Therefore, the participants should be made aware that the scale of the data could allow analysts to connect sequence data to individuals by matching variants in the deposited research data to other data from that person. As well, the public deposition of data in some cases is an irrevocable decision. Once the data are deposited and distributed, it may be impossible to remove the data from all computer servers, should the subject decide to withdraw from the study.

Additionally, participants are informed that once a result is CLIA-certified, that result is placed in the individual’s medical chart of the clinical research institution and may be accessible by third parties. Although there are state and federal laws to protect individuals against genetic discrimination, including GINA, this law has not yet been tested in the courts. This is explained to participants up front at the time of enrollment and a more detailed discussion takes place at the time of results disclosure. To offer additional protection in the event of a court subpoena, a Certificate of Confidentiality has been obtained in the hypothesis-testing and hypothesis-generating protocols. The discussion surrounding privacy and confidentiality is approached in a similar manner in both protocols.

The third issue regarding confidentiality is that MPS can generate many results in each individual and it is highly likely that some, if not all, of the variants detected in one research participant may be present in another research participant (e.g., a parent). This is again a consequence of the scale and breadth of MPS in that the large number of variants that can be detected in each participant makes it exceedingly likely that their relatives share many of these variants and that their genetic risks of rare diseases may be measurably altered. It is important to communicate to the participants that it is likely that such variants can be detected and that they may have implications for other members of the family, and that the consented individuals, or their parent may need to communicate those results to other members of the family.

##### Informed consent approach

The informed consent should include discussion of public deposition of data, the entry of CLIA-validated results into medical records, and the likely discovery of variants with implications for family members.

## Discussion

We describe an approach to the informed consent process as a mutual opportunity for researchers and participants to assess one another’s goals in MPS protocols that employ both hypothesis-generating and hypothesis-testing methodologies. The use of MPS in clinical research requires adaptation of established processes of human subjects protections. The potentially overwhelming scale of information generated by MPS necessitates that investigators and IRBs adapt traditional approaches to consent the subjects. Because nearly all subjects will have a clinically actionable result, investigators must implement thoughtful plan for consent regarding results disclosure, including setting a threshold for the types of information that should be disclosed to the participants.

While some of the informed consent issues for MPS are independent of the study design, others should be adapted based on whether the research study is employing MPS to test a hypothesis (i.e., find the cause of a rare condition in an affected cohort), or to generate hypotheses (i.e., find deleterious or potentially deleterious variants that warrant participant follow-up and further investigation). For example, the health-related attributes of the study cohort (healthy individuals versus disease patients) are likely to influence participants’ motivations and expectations of MPS, and in the case of a disease cohort create the need to dichotomize the genetic variants into primary and secondary. Conversely, issues inherent to MPS technology are central to the informed consent approach in both types of studies. The availability of MPS allows a paradigm shift in genetics research – no longer are investigators constrained to long-standing approaches of hypothesis-testing modes of research. The scale of MPS allows investigators to proceed from genotype to phenotype, and leads to new challenges for genetic and medical counseling. Research participants receiving results from MPS might not present with a personal and/or family history suggestive of conditions revealed by their genotypic variants, and consequently might not perceive their *a priori* risk to be elevated for those conditions.

Participants’ motivations to have whole genome/exome sequencing at this early stage are important to take into consideration in the informed consent process. Initial qualitative data suggest that individuals enroll in the hypothesis-generating study because of altruism in promoting research, and a desire to learn about genetic factors that contribute to their own health and disease risk [23]. Most participants expect that genomic information will improve the overall knowledge of disease causes and treatments. Moreover, data on research participants’ preferences to receive different types of genetic results suggest that they have strong intentions to receive all types of results [16]. However, they are able to discern between the types and quality of information they could learn, and demonstrate stronger attitudes to learn clinically actionable and carrier status results when compared to results that are uncertain or not clinically actionable. These findings provide initial insights into the value these early adopters place on information generated by high-throughput sequencing studies, and help us tailor the informed consent process to this group of individuals. However, more empirical data are needed to guide the informed consent process, including data on research participants’ ability to receive results for multiple disorders and traits.

Participants in both types of studies are engaged in a discussion of the complex and dynamic nature of genomic annotation so that they may make an informed decision about participation and may be aware of the need to revisit results learned at additional time points in the future. As well, we advocate a process whereby investigators retain some latitude with respect to the most serious, potentially life-threatening mutations. While it is mandatory to respect the autonomy of research subjects, this does not mean that investigators must accede to the research subject’s views of these “panic” results. In a paradoxical way, the research participant and the researcher can agree that the latter can maintain a small, but initially ambiguous degree of latitude with respect to these most serious variants. In the course of utilizing MPS technology for further elucidation of the genetic architecture of health and disease, it is imperative that research participants and researchers be engaged in a continuous discussion about the state of scientific knowledge and the types of information that could potentially be learned from MPS. Although resource-intensive, this “partnership model” [2] or informed cohort approach to informed consent promotes respect for participants, and allows evaluation of the benefits and harms of disclosure in a more timely and relevant manner.

## Summary

We have here proposed a categorization of massively-parallel clinical genomics research studies as hypothesis-testing versus hypothesis-generating to help clarify the issue of so-called incidental or secondary results for the consent process, and aid the communication of the research goals to study participants. By using this categorization approach and considering seven important features of this kind of research (Primary versus secondary variant results and the open-ended nature of clinical genomics, Volume and nature of information, Return of individual genotype results, Duty to warn, Length of researcher and participant interaction, Target population, and Privacy and confidentiality) researchers can design an informed consent process that is open, transparent, and appropriately balances risks and benefits of this exciting approach to heritable disease research.

## Competing interests

LGB is an uncompensated consultant to, and collaborates with, the Illumina Corp.

## Authors’ contributions

FMF and JCS drafted the initial manuscript. LGB Organized and edited the manuscript. All authors read and approved the final manuscript.

## Funding

This study was supported by funding from the Intramural Research Program of the National Human Genome Research Institute. The authors have no conflicts to declare.

## Pre-publication history

The pre-publication history for this paper can be accessed here:

http://www.biomedcentral.com/1755-8794/5/45/prepub
